# Total synthesis of (±)-decursivine via BINOL-phosphoric acid catalyzed tandem oxidative cyclization

**DOI:** 10.1038/s41598-021-99064-8

**Published:** 2021-10-07

**Authors:** Prakash T. Parvatkar, Eugene S. Smotkin, Roman Manetsch

**Affiliations:** 1 Department of Chemistry and Chemical Biology, Northeastern University, 102 Hurtig Hall, 360 Huntington Avenue, Boston, MA 02115 USA; 2 Department of Pharmaceutical Sciences, Northeastern University, 102 Hurtig Hall, 360 Huntington Avenue, Boston, MA 02115 USA

**Keywords:** Natural product synthesis, Synthetic chemistry methodology

## Abstract

The synthesis of tetracyclic indole alkaloid (±)-decursivine was accomplished using BINOL-phosphoric acid catalyzed tandem oxidative cyclization as a key step with (bis(trifluoroacetoxy)iodo)benzene (PIFA) as an oxidizing agent. This represents one of the shortest and highest yielding routes for the synthesis of (±)-decursivine from readily available starting materials.

## Introduction

**Figure 1:**
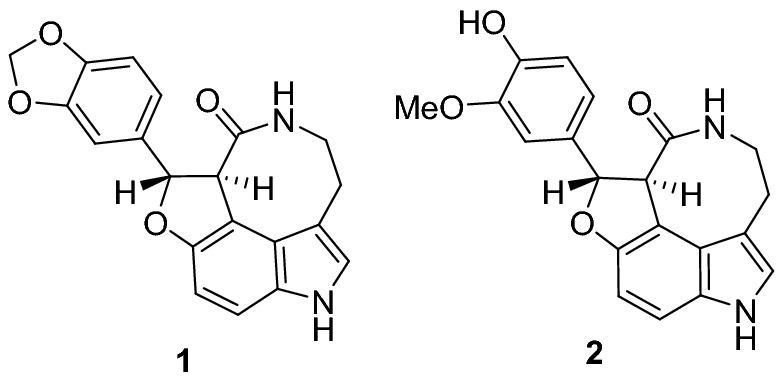
Chemical structures of naturally occurring (+)-decursivine **1** and (±)-serotobenine **2**.

Decursivine **1**, an indole alkaloid, was isolated in the optically active form from the leaves and stems of *Rhaphidophora decursiva Schott* (*Araceae*) by Fong’s group^1^ in 2002. Decursivine is structurally related to serotobenine **2** (isolated as a racemic mixture in 1985 by Sato et al.)^2^ with a unique tetracyclic framework containing a *trans*-dihydrobenzofuran, an indole, and an eight-membered lactam that bridges the indole 3- and 4-positions (Fig. 1). Decursivine **1** exhibits antimalarial activity^1^ against the D6 and W2 isolates of *Plasmodium falciparum* with IC_50_ values of 3.93 and 4.41 µg/mL, respectively, whereas serotobenine **2** exhibits no activity against *Plasmodium falciparum*.

Owing to its novel structural features and potent antimalarial activity, decursivine has been the target of many synthetic efforts over the last decade^3–11^. The first total synthesis of (±)-decursivine was reported in 2007 by Kerr and co-workers^4^. The synthesis was completed in 19 steps with a 3.7% overall yield and featured Diels–Alder / Plieninger indolization reactions as key transformations. In 2011, Jia^5^ and Mascal^6^ independently and simultaneously developed a 4-step total synthesis of (±)-decursivine through a cascade photocyclization/elimination/*O*-Michael addition protocol with overall yields of 47.6% and 53.3%, respectively. In 2013, Jia’s group^7^ extended this cascade via a Witkop photocyclization approach wherein (+)- and (−)-decursivine were obtained with overall yields of 9.5% and 1.6% in 9 and 8 steps, respectively. The first asymmetric total synthesis of (+)-decursivine was developed in 2011 by Li and co-workers^8^ that involves an intramolecular [3 + 2] cycloaddition as the main step with an overall yield of 16.7% over 11 steps. In 2014, Jia’s group^9^ reported the synthesis of (±)-decursivine via a cascade C-H activation/oxidation approach in 4 steps with an overall yield of 19.3%. Subsequently, in 2015, Jia’s group^10^ broadened this cascade approach, implementing C-H activation/oxidation, to synthesize (-)-decursivine in 11 steps with a 6.5% overall yield. More recently, Xia and co-workers^11^ reported an 11-step total synthesis of (+)-decursivine in 2016 using an iron-catalyzed oxidative radical coupling protocol with an overall yield of 17.7%.

## Results and discussion

**Figure 2:**
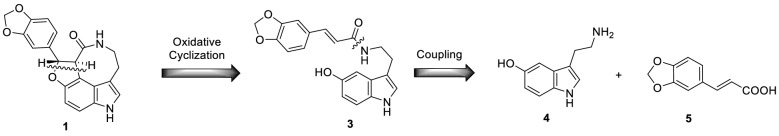
Retrosynthetic pathway of (±)-decursivine **1**.

In continuation of our work towards developing antimalarial heterocyclic compounds^12,13^ and indole-containing natural products^14,15^, we report a 5-step total synthesis of (±)-decursivine, an antimalarial indole alkaloid, from inexpensive and commercially available starting materials. Our retrosynthetic plan is illustrated in Fig. 2. We envisaged that decursivine **1** could be obtained from **3** via tandem oxidative cyclization, which in turn could be prepared by a simple coupling reaction from readily available starting materials, serotonin hydrochloride **4** and 3,4-(methylenedioxy)cinnamic acid **5**.

**Figure 3:**
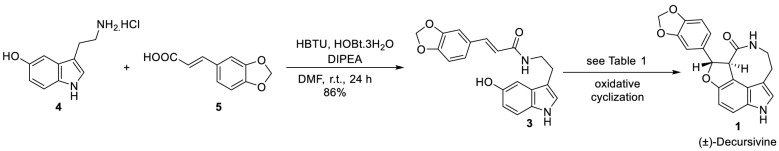
Attempted synthesis of (±)-decursivine **1**.

Our initial efforts towards the synthesis of (±)-decursivine **1** is described in Fig. 3. Coupling of serotonin hydrochloride **4** with 3,4-(methylenedioxy)cinnamic acid **5** using HBTU afforded key intermediate **3**. Direct conversion of **3** into **1** via tandem oxidative cyclization (oxidation through single-electron transfer followed by cycloaddition) was unsuccessful via both photochemical and electrochemical approaches despite varying oxidizing agents and reaction conditions. In many attempts, **3** underwent decomposition (Table 1).

**Table 1:** Tandem oxidative cyclization of compound **3**.

Sr. No.	Reaction conditions	Time (h)	Yield of 1 (%)
1	Ru(bpz)_3_(PF_6_)_2_ (0.1 equiv), (NH_4_)_2_S_2_O_8_ (2 equiv), CH_3_CN, Ar, blue LED, r.t	18	Decomposed
2	Acridinium (0.1 equiv), TBHP (2 equiv), CH_3_CN, Ar, blue LED, r.t	48	Decomposed
3	CAN (2.5 equiv), CH_3_CN, Ar, 0 °C	2	Decomposed
4	Mn(OAc)_3_·2H_2_O (4 equiv), CH_3_CN, Ar, reflux	5	Decomposed
5	PIFA (1.2 equiv), HFIP, Ar, r.t	4	Decomposed
6^a^	Glassy carbon anode, Glassy carbon cathode, 0.1M LiClO_4_, 1.25 V	18	–
7^a^	Platinum anode, Glassy carbon cathode, 0.1MLiClO_4_,1.25 V	10	–

^a^Passivation of electrode observed.

**Figure 4:**
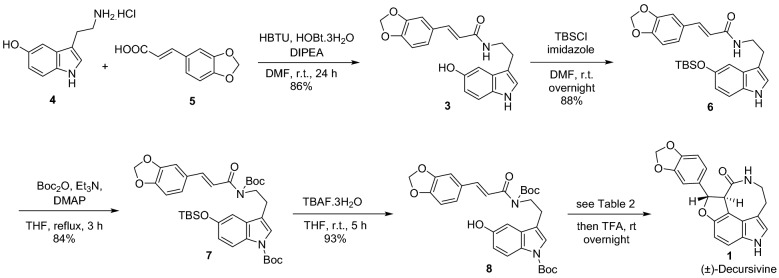
Total synthesis of (±)-decursivine **1**.

These failures motivated us to protect the indole and amide –NH groups (Fig. 4). The hydroxy group of **3** was first protected using TBSCl, then the indole and amide –NH groups were protected by heating a mixture of **6**, Boc_2_O, and DMAP in THF at reflux. Silyl group deprotection of compound **7** by treatment with TBAF yielded hydroxy derivative **8**. With compound **8** on hand, we investigated tandem oxidative cyclization reaction under various conditions (Table 2).

**Table 2:** Tandem oxidative cyclization of compound **8**.

Sr. No.	Reaction conditions	Time (h)	Yield^a^ of 1 (%)
1	I_2_ (0.1 equiv), TBHP (2 equiv), EtOH, Ar, reflux	48	No reaction
2	CAN (2.5 equiv), CH_3_CN, Ar, 0 °C	2	Decomposed
3	Mn(OAc)_3_·2H_2_O (4 equiv), CH_3_CN, Ar, reflux	5	Decomposed
4	KHMDS (1.05 equiv), THF, Ar, 0 °C to r.t	2	Decomposed
5	Ru(bpz)_3_(PF_6_)_2_ (0.1 equiv), (NH_4_)_2_S_2_O_8_ (2 equiv), CH_3_CN, Ar, blue LED, r.t	24	Decomposed
6	[Ir(dtbbpy)(ppy)_2_[PF_6_] (0.1 equiv), BrCCl_3_ (2 equiv), CH_3_CN, Ar, blue LED, r.t	36	Decomposed
7	Acridinium (0.1 equiv), TBHP (2 equiv), CH_3_CN, Ar, blue LED, r.t	48	Decomposed
8	PIFA (1.2 equiv), HFIP, Ar, r.t	6	47
9	PIFA (1.2 equiv), H_3_PO_4_ (0.1 equiv), HFIP, Ar, r.t	4	Trace amounts
10	PIFA (1.2 equiv), (±)-BINOL phosphoric acid (0.05 equiv), HFIP, Ar, r.t	3	74

^a^Isolated yield after column chromatography.

**Figure 5:**
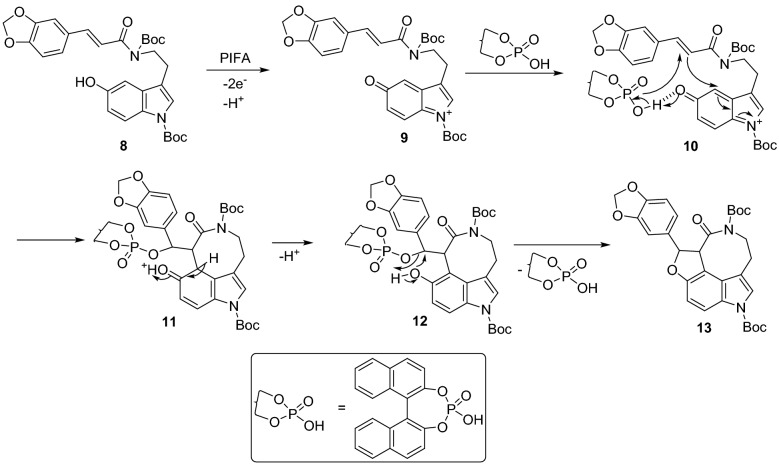
Proposed mechanism of tandem oxidative cyclization.

Oxidation of Boc-protected compound **8** through single-electron transfer, followed by cyclization using different oxidizing agents or photochemical approaches (entries 1–7) did not yield the desired product. Instead, compound **8** underwent decomposition. We then turned our attention towards a two-electron oxidation/cyclization approach using a hypervalent iodine reagent. In the literature, hypervalent iodine has been used for the oxidative [3 + 2] cycloaddition of various phenols and styrenes to yield 2,3-dihydrobenzofuran derivatives^16–20^. Based upon these findings, we treated compound **8** with a hypervalent iodine reagent, (bis(trifluoroacetoxy)iodo)benzene (PIFA), and product **1** was obtained in 47% yield (entry 8). The moderate yield of the product could be due to partial decomposition of the quinone intermediate (formed in situ) before undergoing the cycloaddition. Masson’s group^21^ recently reported the use of chiral phosphoric acid to catalyze the intermolecular oxidative [3 + 2] cycloaddition for the asymmetric synthesis of 3-aminodihydrobenzofurans. With the idea of stabilizing the in situ formed quinone intermediate^21^ via hydrogen bonding, we attempted the [3 + 2] cycloaddition with H_3_PO_4_ (entry 9) as a catalyst. Under these conditions, the reaction was sluggish, forming trace amounts of product that was only observed by LC–MS. Using (±)-BINOL phosphoric acid (entry 10), the reaction was faster and the product was obtained in higher yield. The proposed mechanism for the (±)-BINOL phosphoric acid-catalyzed [3 + 2] cycloaddition is shown in Fig. 5.

PIFA oxidizes **8** to form quinone intermediate **9** which may be stabilized by (±)-BINOL phosphoric acid through hydrogen bonding^21^ to give adduct **10**. Intramolecular cyclization of adduct **10** forms eight-membered lactam intermediate **11** that loses a proton to generate phenolic species **12**. During this process, (±)-BINOL phosphoric acid is released for the next catalytic cycle. Finally, annulation of **12** leads to the formation of 2,3-dihydrobenzofuran containing compound **13** (*N*-Boc-protected decursivine)^8^.

Next, we investigated the use of chiral BINOL phosphoric acid catalysts, shown in Table 3, to perform an asymmetric version of the aforementioned reaction. We employed (*S*)-BINOL phosphoric acid (entry 1) and the bulky (*R*)-3,3’-bis(2,4,6-triisopropylphenyl)-BINOL phosphoric acid (entry 2) to induce chirality during oxidative cyclization. Unfortunately, negligible (˂ 2%) or no enantioselectivity was observed and the racemic product was obtained in 74% and 67% yield, respectively.

**Table 3:** Attempted asymmetric synthesis of decursivine using chiral BINOL phosphoric acid.


Sr. No.	Reaction conditions	Yield^a^ (%)	ee^b^ (%)
1	PIFA (1.2 equiv), (S)-BINOL phosphoric acid (0.05 equiv), HFIP, Ar, r.t., 3 h	74	–
2	PIFA (1.2 equiv), (R)-3,3'-bis(2,4,6-triisopropylphenyl)-BINOL phosphoric acid (0.05 equiv), HFIP, Ar, r.t., 3 h	67	< 2%

^a^Isolated yield after column chromatography.

^b^Determined by HPLC.

## Conclusion

In conclusion, we have developed a concise total synthesis of (±)-decursivine, an antimalarial natural product via a cascade oxidative cyclization using PIFA as an oxidizing agent and (±)-BINOL phosphoric acid as a catalyst with a good overall yield of 43.8%.

## Methods

### General remarks

Reagents and solvents were purchased from commercial suppliers (Fisher Scientific {Hampton, New Hampshire, USA}, Sigma-Aldrich {St. Louis, Missouri, USA}, Strem Chemicals {Newburyport, Massachusetts, USA} and TCI America {Portland, Oregon, USA}) and used without further purification unless otherwise stated. Melting points were recorded on MEL-TEMP laboratory device. ^1^H and ^13^C NMR spectra were recorded at ambient temperature on a Varian Mercury NMR spectrometer (Palo Alto, California, USA) operating at 400 MHz (^1^H NMR) and 100 MHz (^13^C NMR) in the solvent indicated with the signal of the residual solvent (Chloroform-*d* δ 7.26 ppm or Methanol-*d*_4_ δ 3.31 ppm or Pyridine-*d*_5_ δ 8.74, 7.58, 7.22 ppm) as internal standard. Data are reported as follows: chemical shift, multiplicity (s = singlet, d = doublet, dd = doublet of doublets, t = triplet, q = quartet, m = multiplet), coupling constant (Hz) and integration. Thin-layer chromatography (TLC) was performed with silica gel 60 F_254_ pre-coated plates and visualized with exposure to UV light (254 nm) or by iodine staining. Flash chromatography was performed on Biotage (Model: Isolera, Sweden). HPLC analyses were performed using multiwavelength detector (Agilent Technologies 1200 Series). The chiral column used for the HPLC analysis was Lux 5 µm Cellulose-4 (Phenomenex, 250 × 4.6 mm). ESI–MS were recorded on Agilent Technologies 6120 Quadrupole spectrometer (Santa Clara, California, USA). HRMS were recorded on MDS Analytical Technologies AB CSIEX TOF/TOF 5800 spectrometer (Sunnyvale, California, USA). Electrochemical Experiments were performed with an EZstat Pro potentiostat galvanostat (NuVant Systems, Crown Point, IN, USA).

### Synthesis of compound 3

Serotonin hydrochloride **4** (306 mg, 1.44 mmol) and 3,4-(methylenedioxy)cinnamic acid **5** (276 mg, 1.44 mmol) was dissolved in DMF (10 mL). To this solution was added DIPEA (375 µL, 2.15 mmol), HOBt∙3H_2_O (326 mg, 1.72 mmol), and HBTU (655 mg, 1.72 mmol) and the mixture was stirred at room temperature for 24 h. The reaction mixture was diluted with EtOAc (50 mL) and washed with saturated aqueous NH_4_Cl, H_2_O and brine. The organic phase was dried over anhydrous Na_2_SO_4_ and the solvent was removed under reduced pressure. Crude product was purified using flash chromatography on a Biotage Snap Cartridge (KP-Sil 25 g) using a gradient solvent system (40% to 90% ethyl acetate in hexanes) to give product **3** (434 mg, 86% yield). White solid. Mp 96–98 °C. R_f_ = 0.33 (70% ethyl acetate in hexanes). ^1^H NMR (400 MHz, Methanol-*d*_4_) δ 9.97 (s, 1H), 8.09 (s, 1H), 7.42 (d, *J* = 15.7 Hz, 1H), 7.15 (d, *J* = 8.6 Hz, 1H), 7.08–6.83 (m, 4H), 6.79 (d, *J* = 7.9 Hz, 1H), 6.66 (d, *J* = 9.0 Hz, 1H), 6.38 (d, *J* = 15.6 Hz, 1H), 5.95 (s, 2H), 3.54 (d, *J* = 7.5 Hz, 2H), 2.91 (t, *J* = 7.3 Hz, 2H). ^13^C NMR (100 MHz, Methanol-*d*_4_) δ 167.7, 149.9, 149.4, 148.6, 140.2, 129.5, 128.3, 123.9, 123.1, 118.7, 111.5, 111.2, 108.2, 105.9, 102.3, 101.7, 48.1, 47.8, 47.6, 40.3, 25.3. ESI–MS: *m/z* 351 [M + H]^+^. HRMS (MALDI-TOF) calcd for C_20_H_18_N_2_O_4_Na [M + Na]^+^ 373.1159, found 373.1129.

### Synthesis of compound 6

To a solution of compound **3** (296 mg, 0.84 mmol) in DMF (10 mL) was added imidazole (172 mg, 2.53 mmol) and TBSCl (152 mg, 1.01 mmol) and the mixture was stirred at room temperature overnight. Reaction mixture was diluted with EtOAc (50 mL) and washed with saturated aqueous NH_4_Cl, H_2_O and brine. The organic phase was dried over anhydrous Na_2_SO_4_ and the solvent was removed under reduced pressure. Crude product was purified using flash chromatography on a Biotage Snap Cartridge (KP-Sil 25 g) using a gradient solvent system (30% to 80% ethyl acetate in hexanes) to give product **6** (345 mg, 88% yield). White solid. Mp 68–72 °C. R_f_ = 0.47 (70% ethyl acetate in hexanes). ^1^H NMR (400 MHz, Chloroform-*d*) δ 7.91 (s, 1H), 7.51 (d, *J* = 15.5 Hz, 1H), 7.22 (d, *J* = 8.6 Hz, 1H), 7.02 (dd, *J* = 9.4, 2.3 Hz, 2H), 6.98–6.91 (m, 2H), 6.78 (dd, *J* = 8.6, 2.3 Hz, 2H), 6.11 (d, *J* = 15.5 Hz, 1H), 5.98 (s, 2H), 5.61 (s, 1H), 3.71 (q, *J* = 6.4 Hz, 2H), 2.98 (t, *J* = 6.5 Hz, 2H), 0.99 (s, 9H), 0.18 (s, 6H). ^13^C NMR (100 MHz, Chloroform-*d*) δ 166.4, 149.3, 149.2, 148.4, 140.8, 132.3, 129.4, 128.2, 124.1, 123.3, 119.1, 116.5, 111.9, 108.7, 108.4, 106.5, 101.6, 60.7, 40.1, 26.0, 25.5, 18.4, 14.4, -4.2. ESI–MS: *m/z* 465 [M + H]^+^. HRMS (MALDI-TOF) calcd for C_26_H_32_N_2_O_4_SiNa [M + Na]^+^ 487.2024, found 487.2028.

### Synthesis of compound 7

To a solution of compound **6** (331 mg, 0.71 mmol) in THF (20 mL) was added DMAP (87 mg, 0.71 mmol), Et_3_N (396 µL, 2.82 mmol) and Boc_2_O (1.52 g, 6.97 mmol). The reaction mixture was refluxed for 3 h. Solvent was removed under reduced pressure and the residue was diluted with CH_2_Cl_2_ (30 mL) and washed with saturated aqueous NH_4_Cl, H_2_O and brine. The organic phase was dried over anhydrous Na_2_SO_4_ and the solvent was removed under reduced pressure. Crude product was purified using flash chromatography on a Biotage Snap Cartridge (KP-Sil 25 g) using a gradient solvent system (2% to 40% ethyl acetate in hexanes) to give product **7** (398 mg, 84% yield). White solid. Mp 64–66 °C. R_f_ = 0.62 (40% ethyl acetate in hexanes). ^1^H NMR (400 MHz, Chloroform-*d*) δ 7.95 (s, 1H), 7.63 (d, *J* = 15.5 Hz, 1H), 7.41–7.29 (m, 2H), 7.09 (d, *J* = 2.1 Hz, 2H), 7.05 (dd, *J* = 8.0, 1.7 Hz, 1H), 6.87–6.77 (m, 2H), 6.00 (s, 2H), 4.07–3.95 (m, 2H), 2.92 (t, *J* = 7.7 Hz, 2H), 1.64 (s, 9H), 1.48 (s, 9H), 1.00 (s, 9H), 0.22 (s, 6H). ^13^C NMR (100 MHz, Chloroform-*d*) δ 168.9, 153.5, 151.6, 149.5, 148.5, 143.3, 129.9, 124.7, 124.1, 119.8, 117.8, 117.8, 115.9, 109.6, 108.7, 106.9, 101.7, 83.3, 45.1, 28.4, 28.3, 26.0, 24.7, 18.5, -4.2. ESI–MS: *m/z* 565 [M + H-Boc]^+^. HRMS (MALDI-TOF) calcd for C_36_H_48_N_2_O_8_SiNa [M + Na]^+^ 687.3072, found 687.3093.

### Synthesis of compound 8

To a solution of compound **7** (301 mg, 0.45 mmol) in THF (10 mL) was added TBAF·3H_2_O (213 mg, 0.68 mmol) and the mixture was stirred at room temperature for 5 h. Solvent was removed under reduced pressure and the residue was diluted with EtOAc (30 mL) and washed with H_2_O and brine. The organic phase was dried over anhydrous Na_2_SO_4_ and the solvent was removed under reduced pressure. Crude product was purified using Flash chromatography on a Biotage Snap Cartridge (KP-Sil 25 g) using a gradient solvent system (20% to 70% ethyl acetate in hexanes) to give product **8** (232 mg, 93% yield). White solid. Mp 74–78 °C. R_f_ = 0.48 (40% ethyl acetate in hexanes). ^1^H NMR (400 MHz, Chloroform-*d*) δ 7.94 (s, 1H), 7.63 (d, *J* = 15.5 Hz, 1H), 7.41–7.28 (m, 2H), 7.18 (d, *J* = 2.5 Hz, 1H), 7.07 (d, *J* = 1.7 Hz, 1H), 7.05–6.98 (m, 1H), 6.87 (dd, *J* = 8.8, 2.5 Hz, 1H), 6.79 (d, *J* = 7.9 Hz, 1H), 6.00 (s, 2H), 4.02–3.94 (m, 2H), 2.90 (t, *J* = 7.8 Hz, 2H), 1.63 (s, 9H), 1.49 (s, 9H). ^13^C NMR (100 MHz, Chloroform-*d*) δ 169.3, 153.4, 152.0, 148.5, 143.5, 129.8, 124.8, 124.2, 119.7, 117.5, 116.3, 113.4, 108.8, 106.9, 104.7, 101.7, 83.6, 45.2, 28.4, 28.3, 24.8. ESI–MS: *m/z* 452 [M + H-Boc]^+^. HRMS (MALDI-TOF) calcd for C_30_H_34_N_2_O_8_Na [M + Na]^+^ 573.2207, found 573.2204.

### Synthesis of compound (±)-decursivine 1

(i) **Without catalyst**: To a solution of compound **8** (30 mg, 0.05 mmol) in HFIP (5 mL) was added PIFA (28 mg, 0.06 mmol) in one portion under an argon atmosphere and the mixture was stirred at room temperature for 6 h. TFA (20 µL, 0.26 mmol) was then added and the reaction mixture was stirred at room temperature overnight. The reaction was quenched with saturated aqueous NaHCO_3_ and extracted with EtOAc (3 × 20 mL). The combined organic phases were dried over anhydrous Na_2_SO_4_ and the solvent was removed under reduced pressure. The crude product was purified using flash chromatography on a Biotage Snap Cartridge (KP-Sil 10 g) using a gradient solvent system (30% to 90% ethyl acetate in hexanes) to give product **1** (9 mg, 47% yield) as beige solid. Mp ˃250 °C, literature^4^ Mp 260 °C (dec.). R_f_ = 0.15 (50% ethyl acetate in hexanes). ^1^H NMR (400 MHz, Pyridine-*d*_5_) ^1,5,6,8^ δ 12.08 (s, 1H), 8.87 (dd, *J* = 10.3, 4.8 Hz, 1H), 7.49 (d, *J* = 8.6 Hz, 1H), 7.39 (s, 1H), 7.30 (d, *J* = 8.5 Hz, 2H), 7.08 (t, *J* = 8.9 Hz, 2H), 6.94 (d, *J* = 7.9 Hz, 1H), 5.98 (s, 2H), 4.24–4.10 (m, 1H), 3.59 (dd, *J* = 15.8, 3.9 Hz, 1H), 3.22–3.16 (m, 2H). ESI–MS: *m/z* 349 [M + H]^+^. HRMS (MALDI-TOF) calcd for C_20_H_16_N_2_O_4_Na [M + Na]^+^ 371.1002, found 371.1006.
(ii) **With catalyst**: To a solution of compound **8** (30 mg, 0.05 mmol) in HFIP (5 mL) was added (±)-BINOL phosphoric acid (1 mg, 0.0025 mmol) and PIFA (28 mg, 0.06 mmol) in one portion under an argon atmosphere and the reaction mixture was stirred at room temperature for 3 h. TFA (20 µL, 0.26 mmol) was then added and the reaction mixture was stirred at room temperature overnight. The reaction was quenched with saturated aqueous NaHCO_3_ and extracted with EtOAc (3 × 20 mL). The combined organic phases was dried over anhydrous Na_2_SO_4_ and the solvent was removed under reduced pressure. Crude product was purified using flash chromatography on a Biotage Snap Cartridge (KP-Sil 10 g) using a gradient solvent system (30% to 90% ethyl acetate in hexanes) to give product **1** (14 mg, 74% yield). ^1^H NMR and mass data are same as mentioned above and matches with the literature data^1,5,6,8^.

## Supplementary Information

Supplementary Figures.
