# Supplementary material for: Total synthesis of (±)-decursivine via BINOL-phosphoric acid catalyzed tandem oxidative cyclization
Source: Sci Rep. 2021 Oct 7;11:19915. doi: 10.1038/s41598-021-99064-8 (PMC8497573; doi:10.1038/s41598-021-99064-8)
Supplement: Supplementary file 1 — Supplementary Figures. [file 41598_2021_99064_MOESM1_ESM.pdf]

Supporting Information

Total Synthesis of (±)-Decursivine via BINOL-Phosphoric Acid Catalyzed Tandem Oxidative Cyclization

Prakash T. Parvatkar,[1] Eugene Smotkin,[1] Roman Manetsch*[,1,2]

[1]Department of Chemistry and Chemical Biology, Northeastern University, 102 Hurtig Hall, 360 Huntington Avenue, Boston, MA 02115, United States.

[2]Department of Pharmaceutical Sciences, Northeastern University, 102 Hurtig Hall, 360 Huntington Avenue, Boston, MA 02115, United States.

E-mail: r.manetsch@northeastern.edu
Fax: +1-6173738795
Phone: +1-6173736316

^{1}H and ^{13}C NMR Spectra

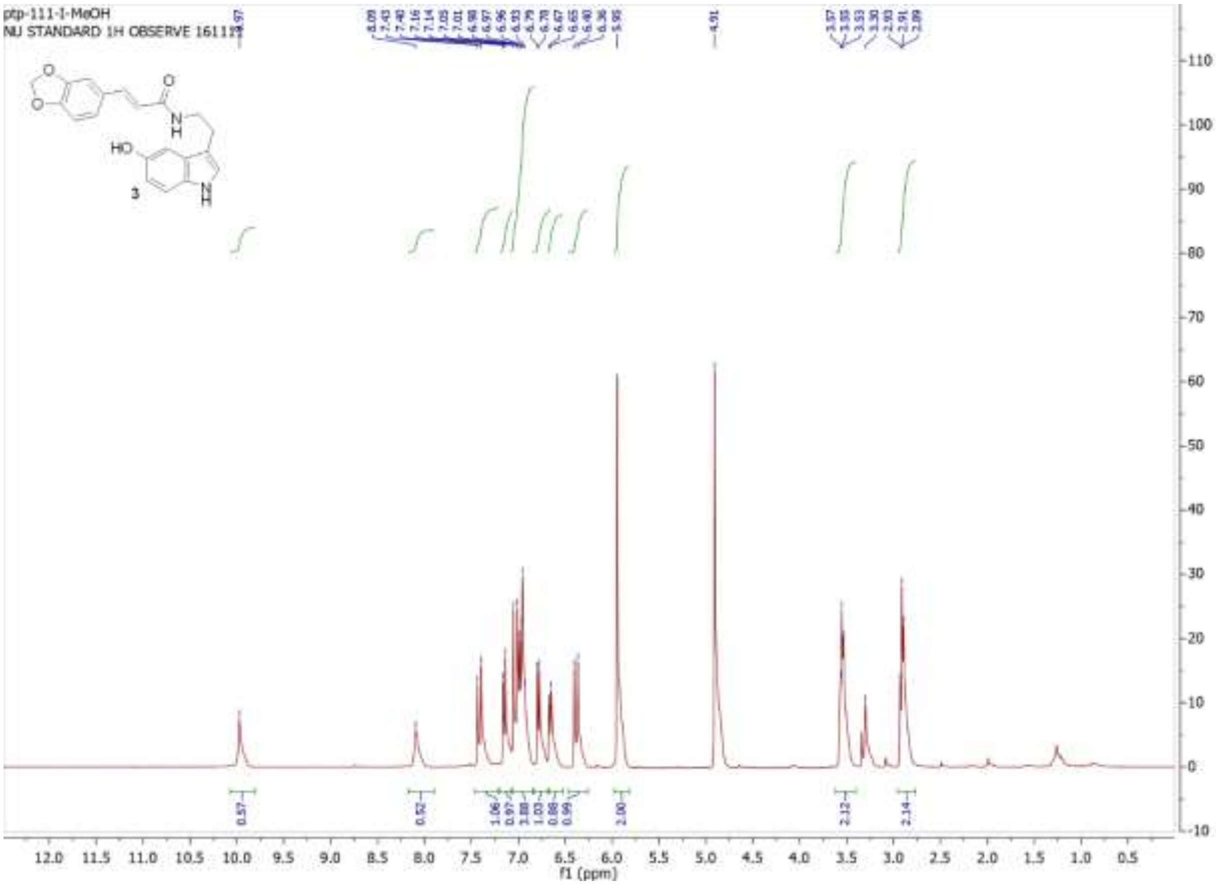

Figure S1: ^{1}H NMR spectrum of Compound **3**.

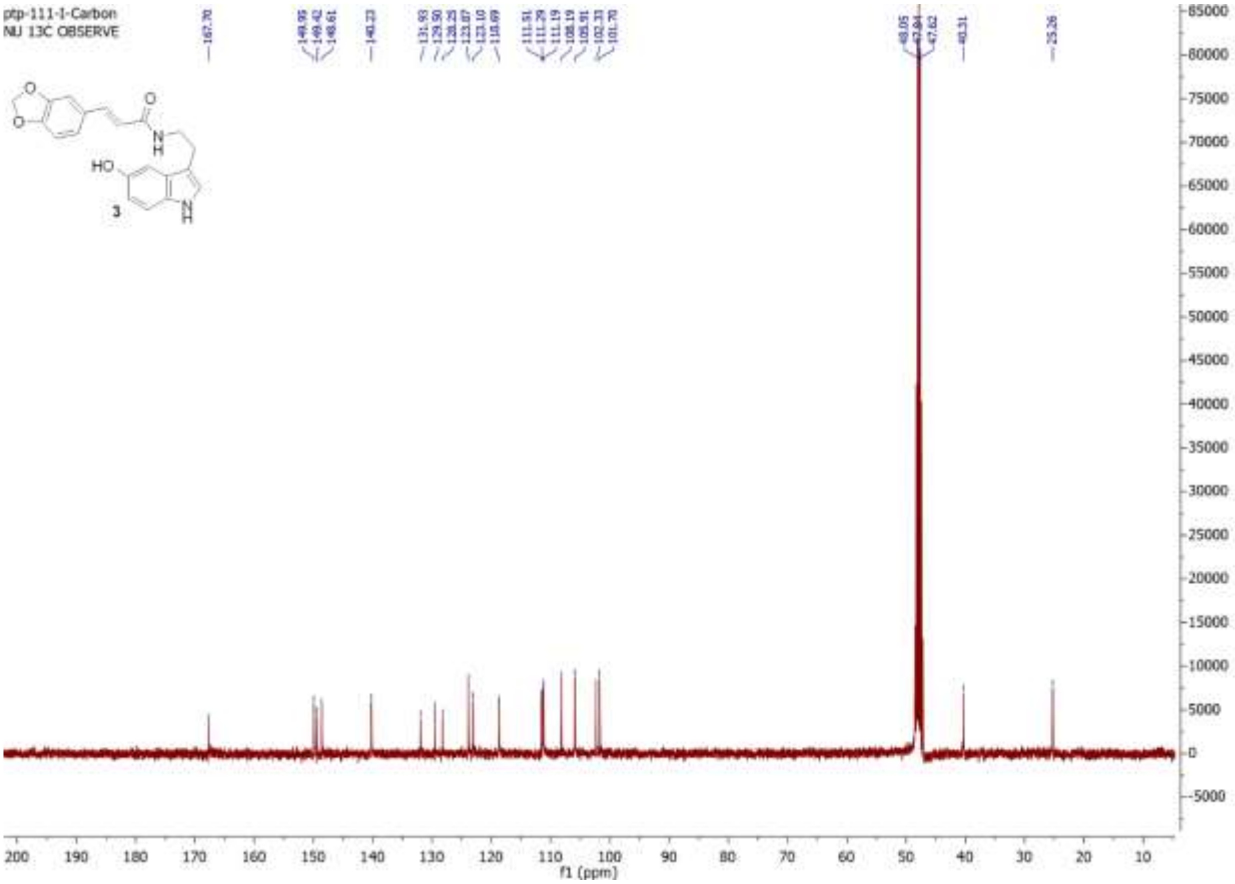

Figure S2: ^{13}C NMR spectrum of Compound **3**.

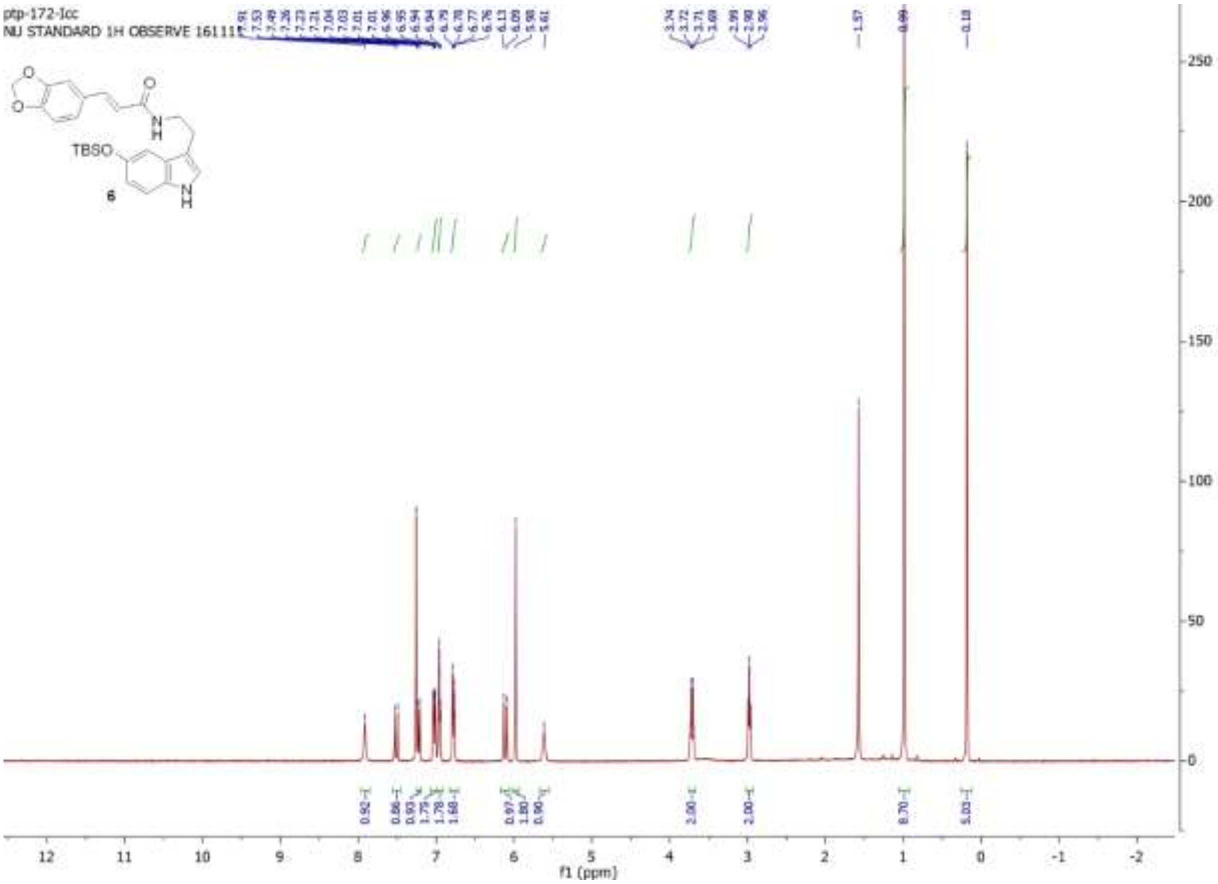

Figure S3: ^{1}H NMR spectrum of Compound **6**.

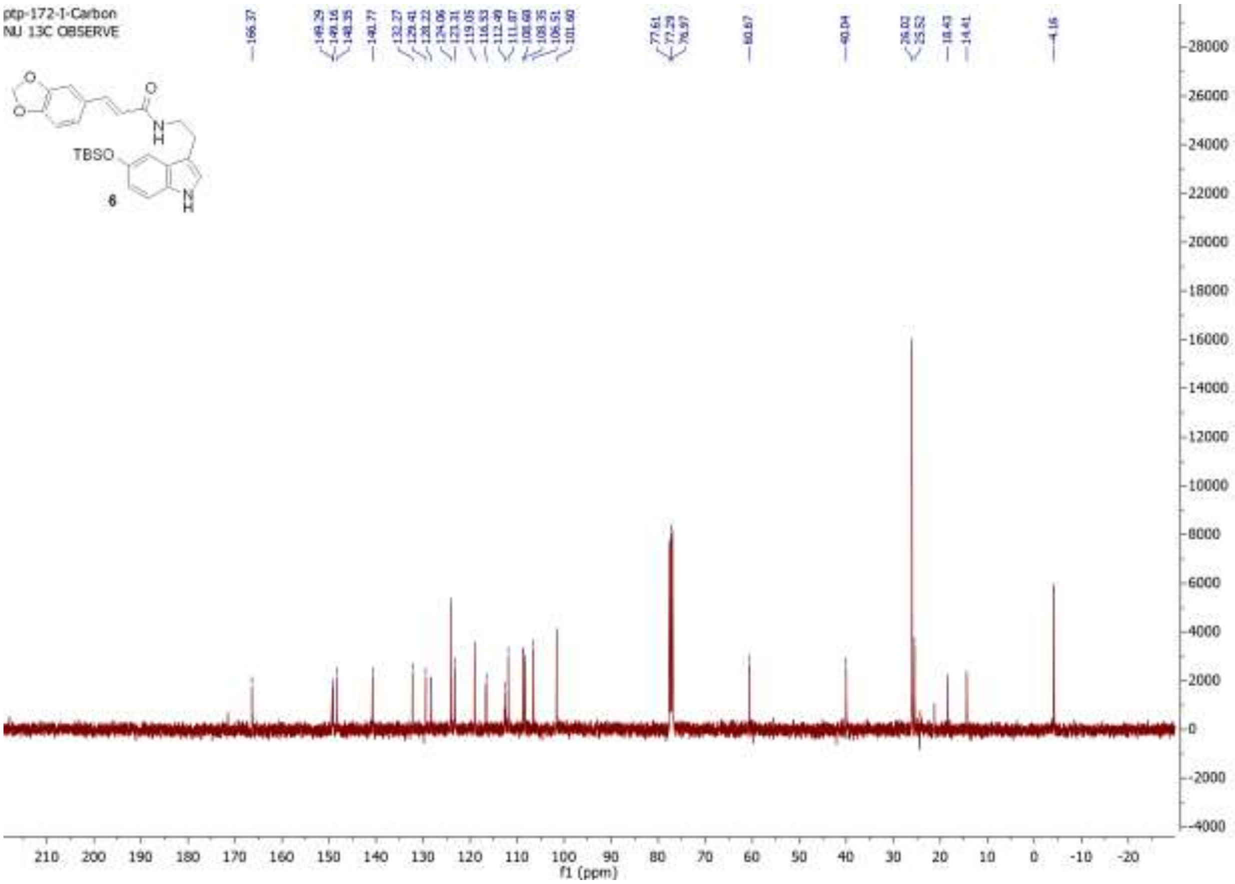

Figure S4: ^{13}C NMR spectrum of Compound **6**.

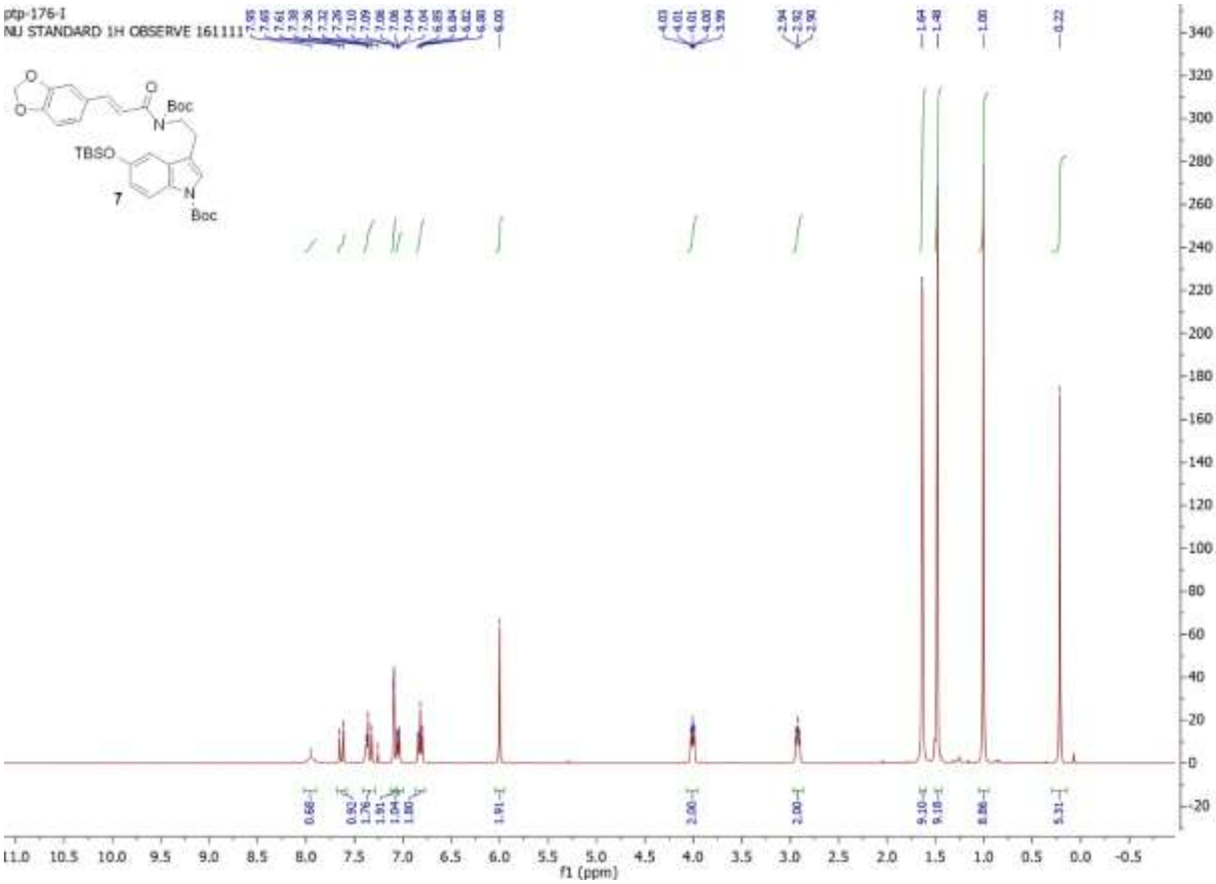

Figure S5: ^{1}H NMR spectrum of Compound **7**.

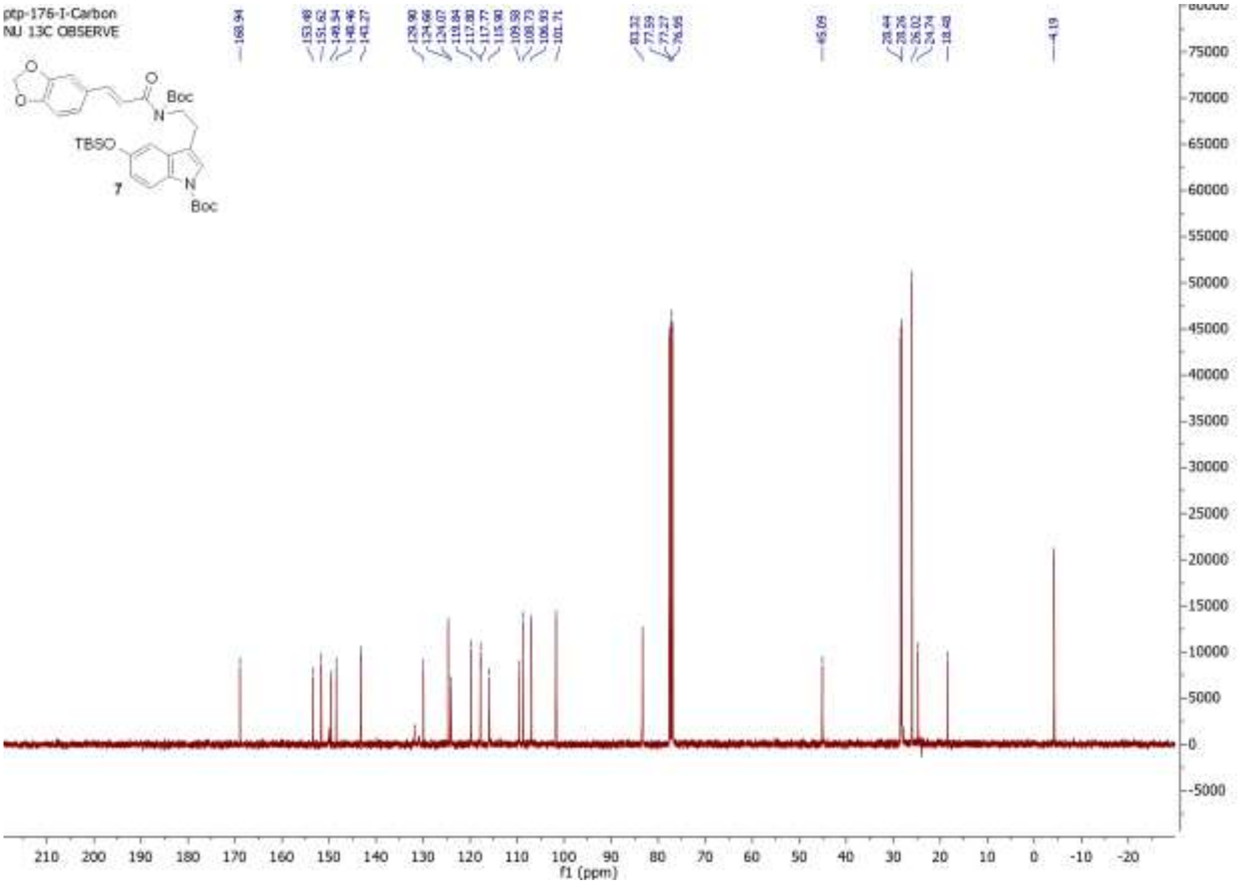

Figure S6: ^{13}C NMR spectrum of Compound **7**.

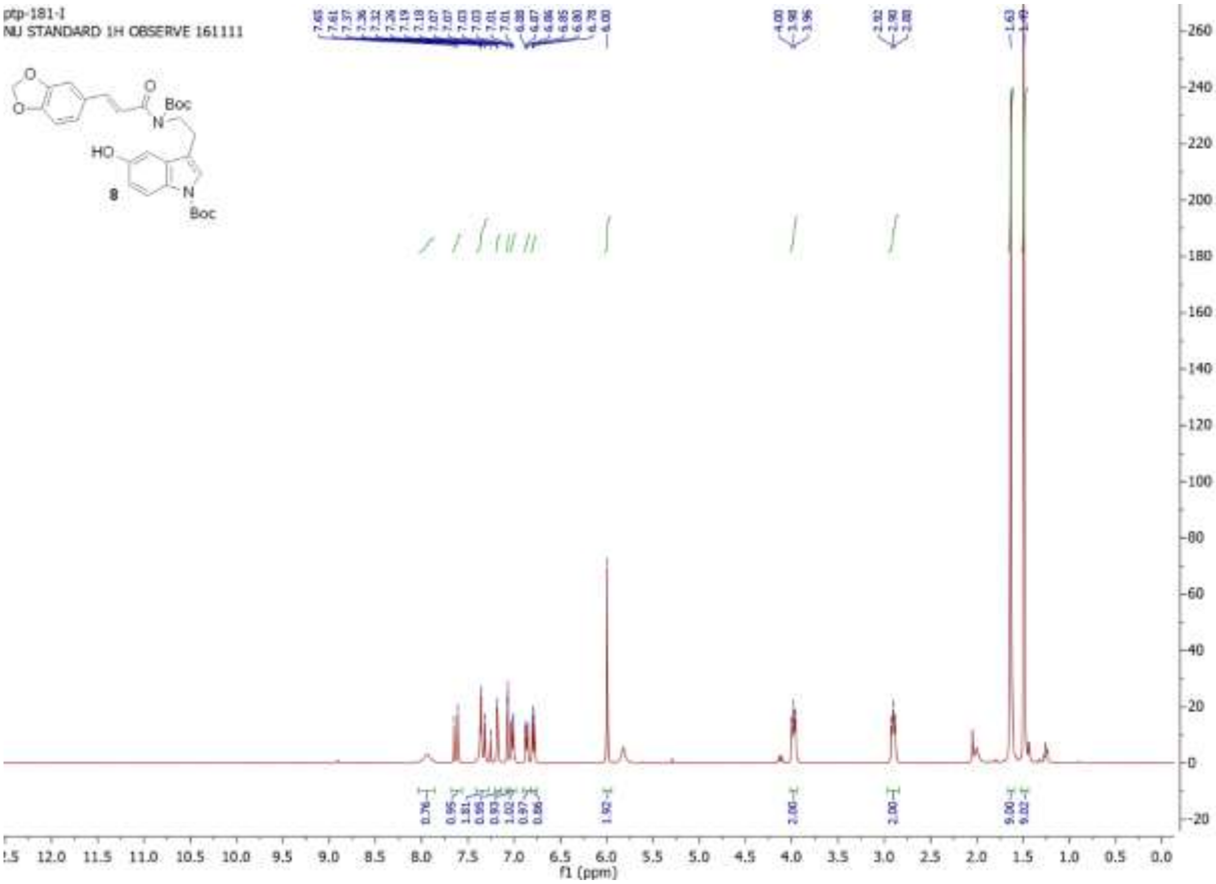

Figure S7: ^{1}H NMR spectrum of Compound **8**.

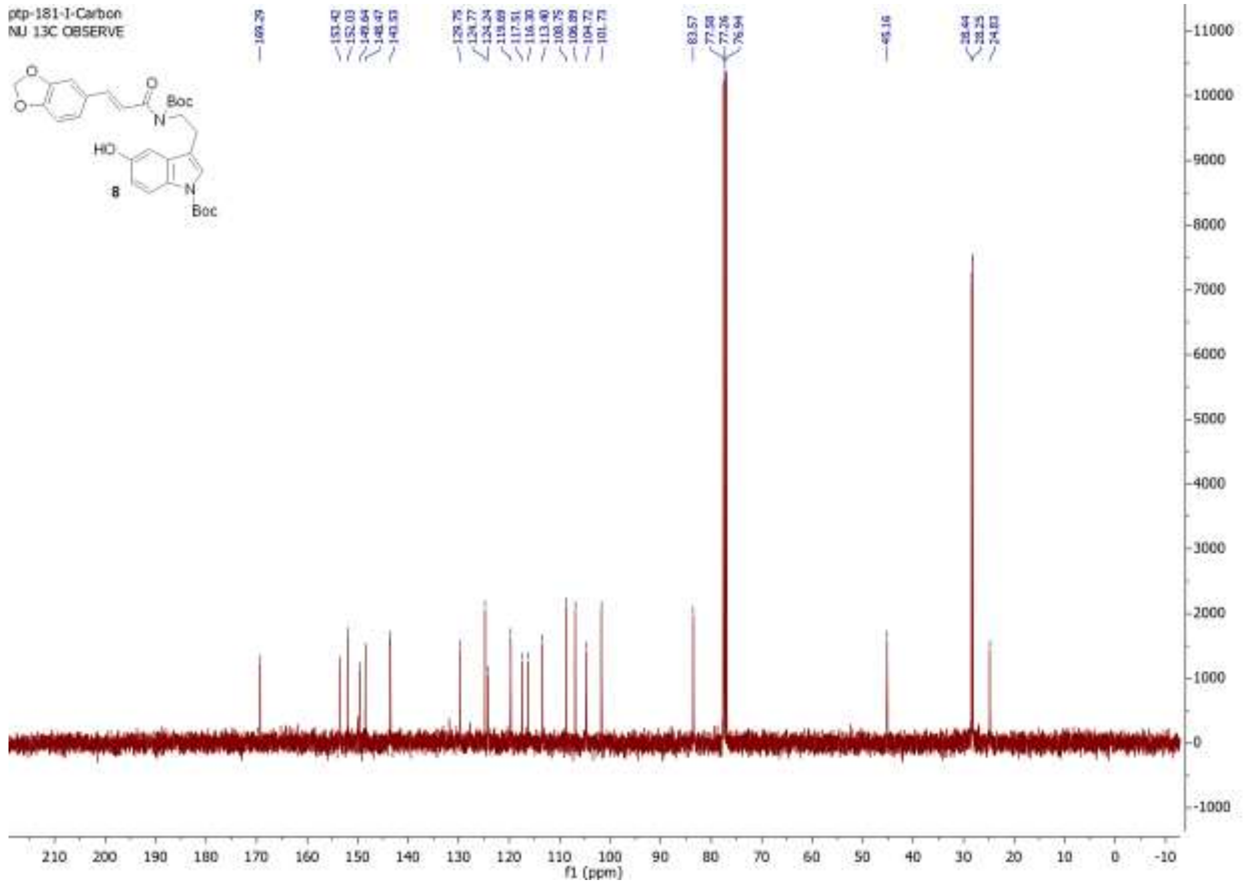

Figure S8: ^{13}C NMR spectrum of Compound **8**.

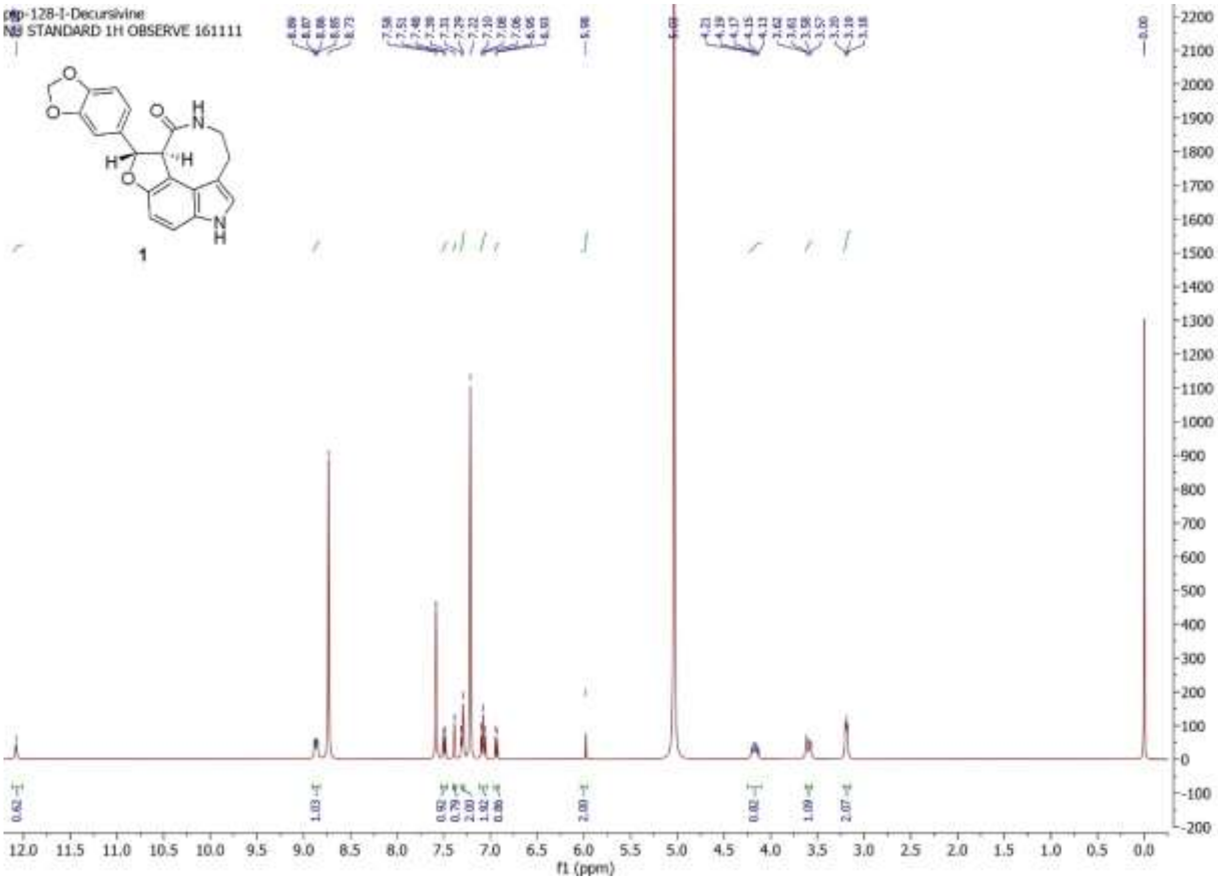

Figure S9: ^{1}H NMR spectrum of Compound **1**.

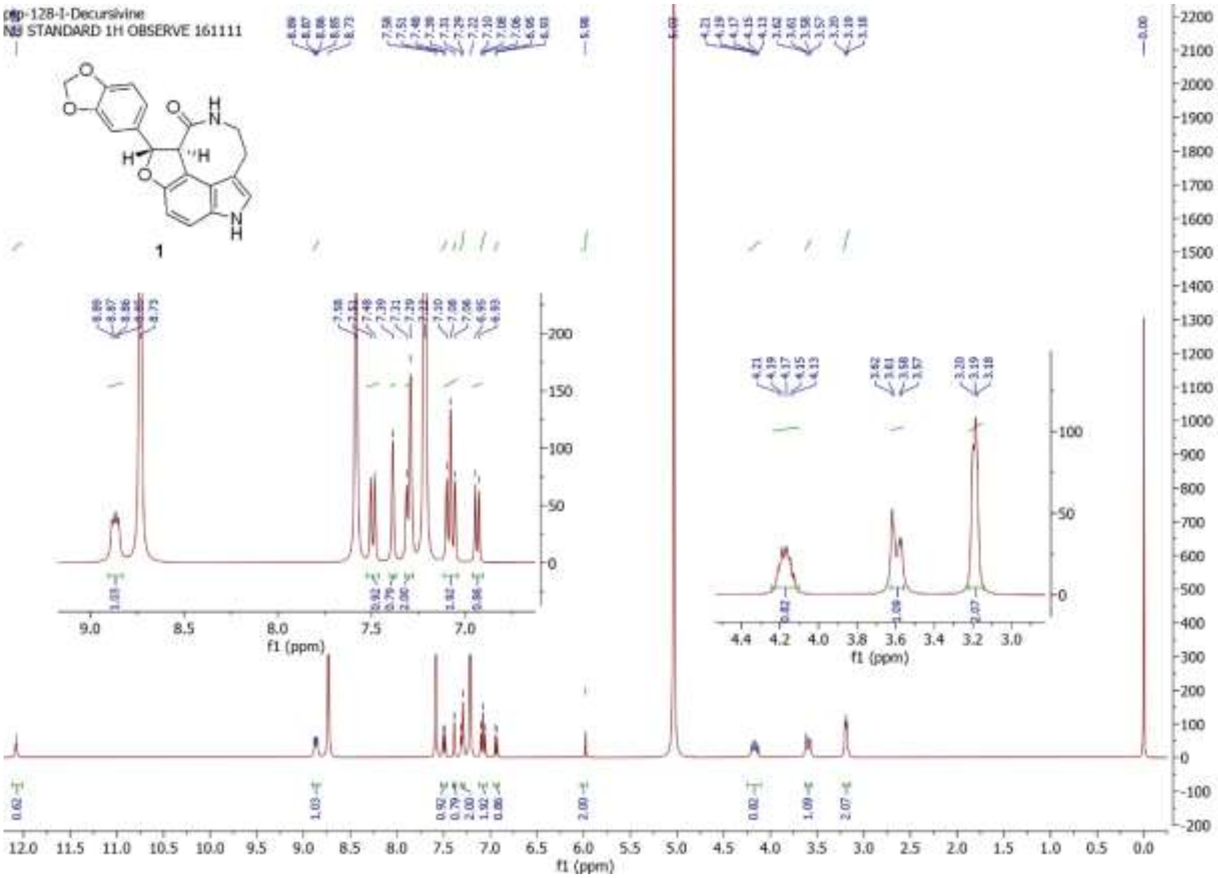

Figure S10: ^{1}H NMR spectrum of Compound **1** with expanded regions.

HPLC Analysis of Compound 1:

Lux 5µm Cellulose-4 chiral column, eluent: H_2O/MeOH (90/10 → 10/90 over 15 min), flow rate: 1mL/min, 25 °C, λ = 280 nm. Compound **1** is racemic containing a pair of enantiomers, t = 8.11 min (one enantiomer) and t = 10.91 min (other enantiomer).

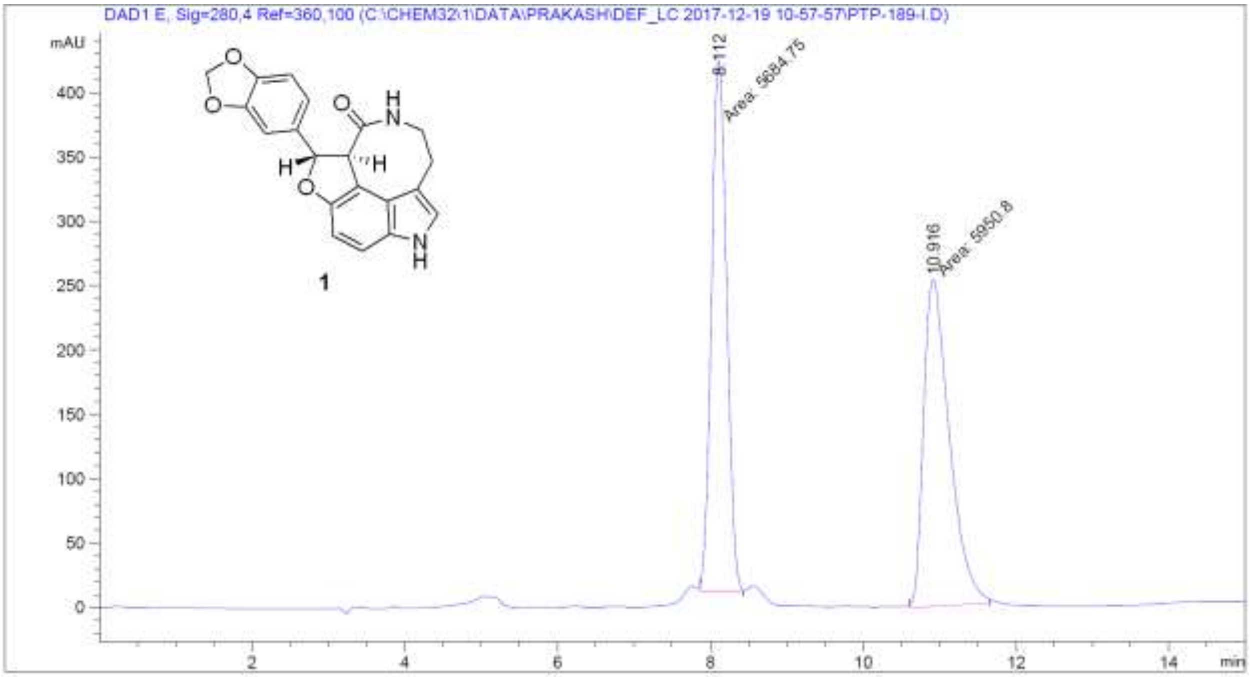

Figure S11: HPLC spectrum of Compound **1**.
